# An emerging allergen: *Cannabis sativa* allergy in a climate of recent legalization

**DOI:** 10.1186/s13223-020-00447-9

**Published:** 2020-06-26

**Authors:** Bradley Jackson, Erica Cleto, Samira Jeimy

**Affiliations:** 1 Department of Paediatrics, Children’s Hospital, London Health Sciences Centre, London, ON Canada; 2 Schulich School of Medicine and Dentistry, Western University, London, ON Canada; 3 Division of Clinical Immunology and Allergy, Department of Medicine, Western University, London, ON Canada; 4 B3-112, St. Joseph’s Healthcare London, 268 Grosvenor Street, London, ON N6A 4V2 Canada

**Keywords:** *Cannabis sativa*, Allergy, Marijuana

## Abstract

Considering its recent legalization in Canada, the health implications of *Cannabis sativa* exposure, including allergy, are coming to the forefront of medical study and interest. *C. sativa* allergy is an issue that affects recreational users of the substance, processors, agricultural workers, and contacts of *Cannabis* aeroallergens and secondhand product. Allergies to *C. sativa* are heterogenous and span the spectrum of hypersensitivity, from dermatitis to rhinoconjunctivitis to life-threatening anaphylaxis. Due to its recent legalization, sensitized individuals will have increasing exposure from direct contact to agricultural pollens. Diagnosis and treatment of *Cannabis* allergy are developing fields that are already showing promise in the identification of culprit antigens and the potential for immunotherapy; however, much responsibility still falls on clinical diagnosis and symptom management. Hopefully, given the current explosion of interest in and use of *Cannabis*, *C. sativa* allergy will continue to garner awareness and therapeutic strategies.

## Background

*Cannabis sativa* allergy is a hypersensitivity that has recently been gaining relevance and is of particular interest due to recent legalization in Canada. Approximately 17% of Canadians, and 27% of those 25–24 years old, report *Cannabis* use within the past 3 months [1]. *Cannabis sativa* allergy is expected to increase as a consequence of legalization due to increased exposure. Additionally, as legal and stigma-related barriers to use subside, an unintentional side effect of legalization may be increased reporting of current suspected cases of *Cannabis* allergy. Given the potential for increases in existing and reported allergic reactions to *Cannabis*, building an understanding of *C. sativa* allergy spectrum, diagnosis, and treatment will be important moving forward.

The purpose of this article is to provide an overview of the current understanding of *Cannabis* allergy and place it within a Canadian context. This article also highlights that exposure extends beyond recreational use and includes second-hand exposure, ingestion, aeroallergen contact, and cutaneous contact.

## Spectrum of *C. sativa* allergy

*Cannabis* is a complex genus of dioecious, annual, wind-pollinated herbs that diverged from *Humulus*—a small genus that includes *H. lupulus*, whose bitter female flowers form the hops used to flavor beer—approximately 27.8 million years ago [2]. *Cannabis* is among humanity’s oldest crops with records of its use for food, fiber, medicine, and inebriation dating back over 6000 years. Despite its long history of use, its taxonomy remains disputed, with some suggesting a monotypic classification with several subspecies of *C. sativa* [2], and others suggesting three distinct species (*C. sativa*, *C. indica*, and *C. ruderalis*) [3, 4]. The biochemistry of *Cannabis* is similarly complex, with at least 118 cannabinoids and 489 described constituents, the most well know and psychoactive of which being tetrahydrocannabinol (THC) and cannabidiol (CBD) [5]. “Indica” varieties of *Cannabis* tend to have a higher THC content, and higher THC to CBD ratio than “sativa” varieties [2]. “Indica” varieties are known for a more mellow high and a terpenoid profile with an acrid, skunk smell, whereas “sativa” varieties are known for a more exciting high and a sweet, herbal aroma [2]. However, these strains are heterogeneous with genome-wide variability that is not limited solely to the genes involved in THC and CBD production [4].

Study of specific culprit *Cannabis* allergens is still in its infancy. A handful of IgE immunoblot experiments, summarized in Table 1, have identified several potential allergens. Of these, the *Cannabis* non-specific lipid transfer protein (nsLTP), Can s 3, was the first identified and is the best studied [6]. Thaumatin-like protein (TLP), ribulose-1,5-bisphosphate carboxylase oxygenase (RuBisCO), and oxygen evolving enhancer protein 2 have also been recognized as potential sensitizing allergens in *Cannabis* allergy [7, 8].

**Table 1:** Summary of possible *Cannabis* allergens

Molecular weight	Genbank nucleotide	Genbank protein	Description	Study
9 kDa	HE972341.1	CCK33472.1	Lipid transfer protein precursor, partial (chloroplast)	Gamboa et al. [6]
10 kDa	HE972341.1	P86838.1	Non-specific lipid-transfer protein	Larramendi et al. [7]
38 kDa	XM_030636673.1	XP_030492533.1	Thaumatin-like protein 1b	
53 kDa	JP454288.1	YP_009123081.1	Ribulose 1,5-bisphosphate carboxylase/oxygenase large subunit (chloroplast)	Nayak et al. [8]
54 kDa	JP462165.1	YP_009123080.1	ATP synthase CF1 beta subunit (chloroplast)	
29 kDa	JP475070.1	XP_030482568.1	Oxygen-evolving enhancer protein 2, chloroplastic	
49 kDa	JP458088.1	XP_030492156.1	Ribulose bisphosphate carboxylase/oxygenase activase, chloroplastic isoform X2	
52 kDa	JP451043.1	XP_030504809.1	Ribulose bisphosphate carboxylase/oxygenase activase 2, chloroplastic-like	
48 kDa	JP450816.1	XP_030507192.1	Glutamine synthetase leaf isozyme, chloroplastic	
51 kDa	JP458176.1	PON58274.1	Phosphoglycerate kinase (Trema orientale)	
47 kDa	JP473302.1	XP_030489218.1	Fluoride export protein 2-like isoform X1	
48 kDa	JP452228.1	PON90495.1	Glyceraldehyde-3-phosphate dehydrogenase, type I (Trema orientale)	

**Fig. 1:**
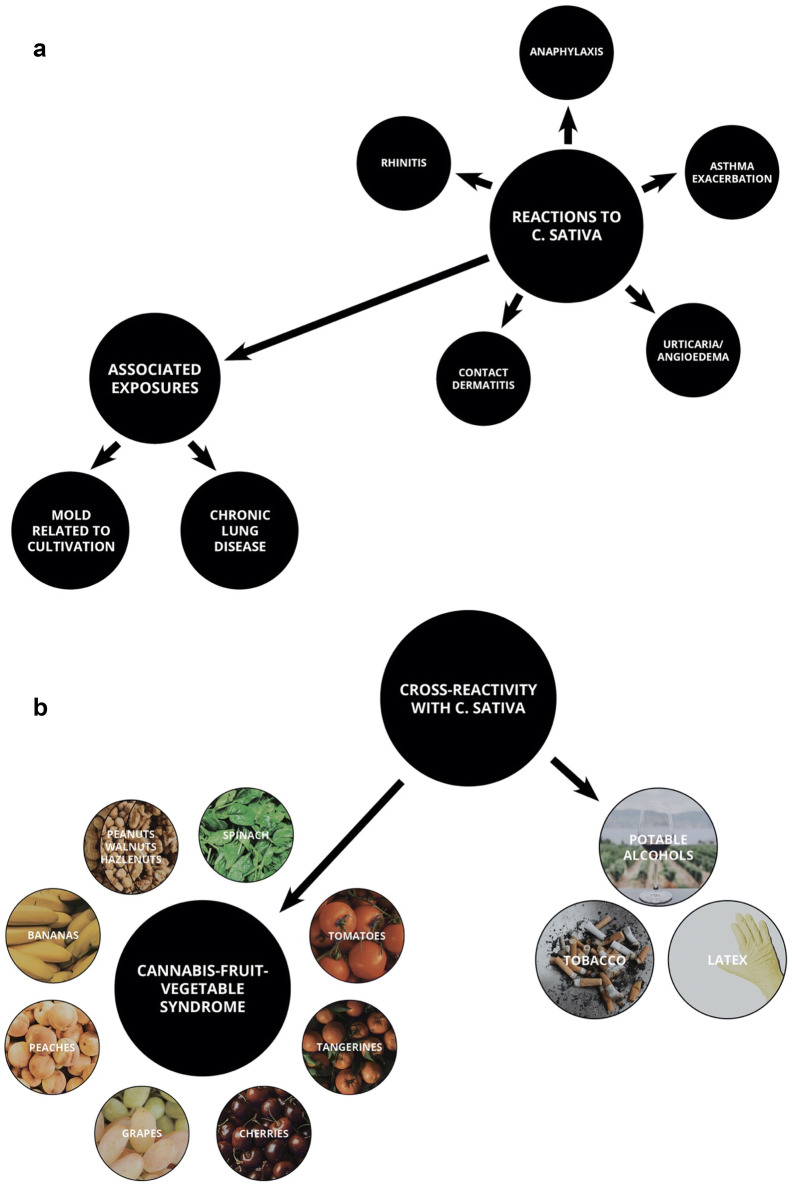
**a** Indicates the different types of allergic reactions and associated exposures to *Cannabis sativa* (*C. sativa*). **b** Shows cross-sensitizations between *C. sativa* and fruits, vegetables, tobacco, alcohol, and latex

*Cannabis* sensitivity spans the spectrum of allergic response. As an aeroallergen, *Cannabis* pollen has been implicated in allergic rhinitis, allergic keratoconjunctivitis, hypersensitivity pneumonitis, and exacerbations of asthma symptoms [9] (Fig. 1a). Additionally, patients may experience cutaneous reactions in the form of generalized pruritus, contact urticaria, and angioedema. A case of occupational contact urticaria was reported in a forensic sciences technician who had regular occupational contact with *Cannabis* for a period of 2 years. She was neither a recreational user, nor an atopic or dermatographic individual, suggesting sensitization specifically from repeated handling [10]. Erythema multiforme (in one case report) has also been associated with recreational consumption [11]. This individual experienced the eruption of vesicobullous, scaled, and targetoid rash on his distal extremities which progressed proximally to his trunk within a two-week period, waxing and waning synchronously with his use of *Cannabis* [11]. Anaphylaxis to *C. sativa* with hempseed ingestion, smoking, and injection have also been reported [12–14].

*Cannabis* has reasonably common, expected, but undesirable physiologic effects (conjunctival injection, sinus tachycardia, orthostatic hypotension, anxiety or panic reactions, dysphoria). It is important to not ignore or mis-attribute similar symptoms when the index of suspicion for a serious reaction or anaphylaxis is high [9].

*Cannabis* consumption also carries a risk to immunosuppressed patients in the form of microbiological contaminants, particularly when inhaled. Aspergillus has been isolated repeatedly from *Cannabis* samples [15, 16]. In one observational study, a majority of *Cannabis* users had antibody evidence of Aspergillus exposure compared to a minority of abstinent controls [17]. Furthermore, cases of pulmonary aspergillosis have been linked to contaminated Cannabis use in immunosuppressed populations [16, 17]. Fungal spores resist destruction from smoking and vaporization [18]. Thus, hypersensitivity and immunosuppression are clinically relevant states with regard to *Cannabis* consumption.

## Sensitization to *C. Sativa*

Sensitization to *Cannabis* can occur via inhalation, cutaneous exposure, ingestion, and secondhand exposure, and can occur in recreational users and occupational handlers. Specifically, sensitization and reactions have been seen with smoking, consuming, injecting, and handling *Cannabis* plants, the latter being most germane to industrial workers [19–21]. As the *Cannabis* agricultural industry grows, *C. sativa* may also become a significant aeroallergen. Indeed, Canada’s first large-scale commercial outdoor Cannabis farm began operations in mid-summer 2019 [22]. The potential role of *Cannabis* pollen as an aeroallergen has long been realized in agricultural regions. For example, in Nebraska, peak season pollen counts show *Cannabis* comprising 36% of the total airborne burden, and additionally correlating with a skin-test positive allergic symptom surge during mid to late August [23].

In light of this increase in *Cannabis* aeroallergen, we may also begin to see an increase in Cannabis-fruit/vegetable syndrome. As with other forms of food-pollen or oral allergy syndrome, Cannabis-fruit/vegetable syndrome is thought to occur due to structural homology and antigenic similarities between nsLTPs in *C. sativa* and those in cherry, tangerine, peach, tomato, hazelnut, latex, and tobacco (Fig. 1b), resulting in cross-sensitivity and reaction to consumption of these products [7, 9, 19]. However, unlike birch pollen-related food-pollen syndrome, Cannabis-fruit/vegetable syndrome may cause more severe symptoms (including anaphylaxis to previously tolerated fruit). Sensitization is bidirectional; i.e. sensitization to an nsLTP in fruits can cause subsequent sensitization to Cannabis [7, 19, 20]. Thus, a variety of exposure routes exist for *C. sativa* sensitization, and these sensitizations may be primary or cross-reactive.

## Diagnosis of *C. sativa* allergy: an evolving practice

Clinical history is the cornerstone of diagnosing *Cannabis* hypersensitivity. As with any other allergic presentation, a complete history will include a detailed review of the presenting suspected reaction (Table 2). The history should also include a thorough review of atopic history, medical history, medications, social history including recreational and occupational exposures, and family history including atopy and asthma. With respect to diagnostic testing, the “gold standard” allergen challenge may not be appropriate in *Cannabis* allergy. Although Canadian law would permit access to and use of the substance unlike many regions, there is dispute regarding expected reaction phenotypes, particularly regarding varied and paradoxical lower airway response [20]. Thus, *Cannabis* graded challenge is not yet a viable, routine diagnostic option. Epicutaneous testing is currently not standardized for *C. sativa*. Skin testing described in current literature is heterogenous and requires the suspension of marijuana buds, leaves, and/or flowers to be produced and administered by the allergist [20]. In vitro assays of serum specific IgE (sIgE), cytometric basophil activation (BAT), and basophil histamine release using crude extracts, purified components and recombinantly expressed allergens have shown promising results, but remain commercially unavailable [20, 21, 24, 25].

**Table 2:** Suggested prompts for a history of presenting suspected reaction to a *C. sativa* product

Suggestions for characterizing the history of a possible presenting reaction to *C. sativa*
Symptoms
Cutaneous (urticaria, contact dermatitis, etc.)
Gastrointestinal (vomiting, diarrhea, abdominal pain, etc.)
Respiratory (wheeze, cough, dyspnea, etc.)
Oropharyngeal/mucosal/conjunctival (nasal obstruction, palatal pruritis, eye pruritis, nasal discharge, etc.)
Other, as described or suspected by patient and clinician
Timeline of reaction
Chronological relation to suspected exposure (immediate vs. delayed)
Course of development of symptoms
Duration of symptoms
Frequency of symptoms
Dependency on exposure
Nature of exposure
Suspected allergen(s)
Route of exposure (oral, smoked, ingested, contact, etc.)
Dose dependency
Form (processed, whole plant, oil, etc.)
Reproducible
Exacerbating factors (alcohol, exercise, other known allergens present)

Adapted from consultation template prompts from the Division of Clinical Immunology and Allergy at St. Joseph’s Healthcare in London, ON

The isolation of specific *Cannabis* antigens will facilitate standardized skin prick and serum IgE testing. Recently, Decuyper et al compared specific IgE (sIgE) testing to hemp, sIgE to a recombinant Can s 3 (rCan s 3) protein, BAT to the same rCan s 3, and skin prick testing with a Can s 3 antigen-rich extract in diagnosing Cannabis allergy [20]. The Can s 3 extract, which is not commercially available, was prepared for study using methods previously described for isolating nsLTPs from tomato, with total protein quantification using Micro BCA Protein Assay [20, 24, 26]. The results of the comparison suggested that Can s 3 is the superior antigen for testing, and that skin prick and sIgE testing are effective and practical, with respective sensitivities of 72% and 81% and specificities of 63% and 87% [20]. While promising, the authors address the clear issue of lack of commercial availability of these extracts. They suggest that, with current clinical limitations, a sIgE to hemp (which is currently available from Thermo Fisher) may be appropriate for diagnosis as only 18% of Cannabis sensitized individuals have negative IgE to hemp. However, it would still be ideal that a commercially available Can s 3 extract become available.

## Treatment of *C. sativa* allergy

The only proven, currently available treatment for *Cannabis* allergy is avoidance. However, when avoidance is impossible, treatment of *C. sativa* allergy is identical to that of other allergens: based on the index reaction to the substance. Treatment with antihistamines, intranasal corticosteroid sprays, and ophthalmic antihistamine drops can provide symptom relief [9]. All individuals with anaphylactic allergies should carry auto-injectable epinephrine. Treatment for Cannabis-fruit-vegetable syndrome is also dependent on avoidance.

Promising but limited case reports suggest future directions for the treatment of *Cannabis* allergy. For example, Engler et al. described an occupationally exposed individual with anaphylaxis to *Cannabis* who was successfully treated for with Omalizumab therapy [27]. Kumar et al. successfully implemented a perennial subcutaneous immunotherapy schedule that reduced a patient’s symptoms of allergic rhinitis and asthma during *Cannabis* pollen season [28]. This was delivered as subcutaneous, twice-weekly doses starting with 1:5000 weight/volume of diluted antigen, beginning at 0.1 mL and increasing by 0.1 mL per injection to a target maintenance dose of 1 mL of 1: 50 antigen concentration per month for 1 year [28].

Hopefully, in light of the rise of *C. sativa* use and agriculture, desensitization protocols will become available for sensitive patients as demand increases. Nonetheless, avoidance and traditional methods of managing allergic reactions continue to be the basis of treatment for *Cannabis* allergy.

## Conclusion

The legalization and accessibility of *Cannabis sativa* in Canada has created a renewed interest in the health implications of its use, including allergic and immunologic consequences. This brief review has highlighted the diversity of sensitization routes and reactions to the plant, emphasizing the heterogenous presentation of *Cannabis* allergy. In addition, this article has underscored the fledgling nature of available testing and treatment options for *C. sativa* allergy. There have been recent, exciting advancements in isolation of culprit allergens and clinical testing, although these are not yet applicable to general office use. At the moment, there are existing practical suggestions for diagnosing and treating *C. sativa* allergy, which will hopefully evolve in the coming years as Can s 3 preparations and immunotherapy schedules mature and become commercially available. However, currently, a detailed allergy history with adjunct hemp sIgE testing are the cornerstones of diagnosis, and avoidance (in combination with standard symptomatic treatment) is the mainstay of treatment.

## Data Availability

Not applicable.

## Abbreviations

*C. sativa*: *Cannabis sativa*
LTP: Lipid transfer protein
Ns: Non-specific
TLP: Thaumatin-like protein
sIgE: Serum immunoglobulin E
BAT: Basophil activation testing
